# A Case of Noncommunicating Hydrocephalus Presenting as Isolated Hyposmia

**DOI:** 10.1002/ccr3.72802

**Published:** 2026-06-04

**Authors:** Bartels Walker, Siddharth A. Patel, Caitlin Jackson, Graham M. Strub

**Affiliations:** ^1^ University of Arkansas for Medical Sciences College of Medicine Little Rock USA; ^2^ Arkansas Children's Hospital Little Rock USA; ^3^ Department of Otolaryngology‐Head and Neck Surgery University of Arkansas for Medical Sciences Little Rock USA

**Keywords:** anosmia, hydrocephalus, hyposmia, olfactory dysfunction

## Abstract

Olfactory dysfunction presents in combination with various different symptoms and can be attributed to a range of etiologies, including sinonasal disease, post‐viral infection, post‐traumatic injury, age‐related decline, and less commonly intracranial structural abnormalities and genetic conditions. Here we report a rare case of noncommunicating hydrocephalus identified following evaluation of an adolescent female presenting with longstanding hyposmia. Etiology was identified on MRI, and surgical correction with a third ventriculostomy led to improvement in olfaction.

## Key Clinical Message

Olfactory dysfunction can be a subtle manifestation of underlying intracranial pathology. This case illustrates that noncommunicating hydrocephalus, though rare, should be considered in the differential diagnosis of hyposmia, as timely neuroimaging and surgical intervention may result in symptom resolution.

## Graphical abstract

Noncommunicating hydrocephalus causing hyposmia.

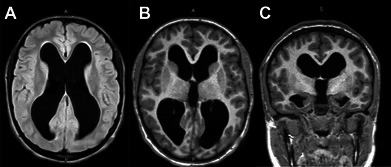

## 1 Introduction

Noncommunicating hydrocephalus, or obstructive hydrocephalus, is caused by the abnormal accumulation of cerebral spinal fluid (CSF) within the ventricles of the brain due to obstruction of CSF flow [1]. This buildup of pressure can damage neural tissue, leading most commonly to headache, visual dysfunction, nausea, and vomiting. While there are many clinical presentations of noncommunicating hydrocephalus, it is less commonly associated with isolated anosmia [2].

Anosmia and hyposmia are terms used to describe complete or partial failure of the sense of smell. Olfactory dysfunction can be a nonspecific finding in a range of conditions, from sinonasal disorders to Parkinson's disease [3, 4], or following viral infections such as COVID‐19 or influenza [5]. This can be a subtle finding, often overshadowed by more distressing symptoms such as headache, nausea, or vision changes.

Here we present a case of isolated hyposmia in an adolescent female that led to neuroimaging and a subsequent diagnosis of noncommunicating hydrocephalus requiring neurosurgical intervention.

## 2 Case History/Examination

**FIGURE 1:**
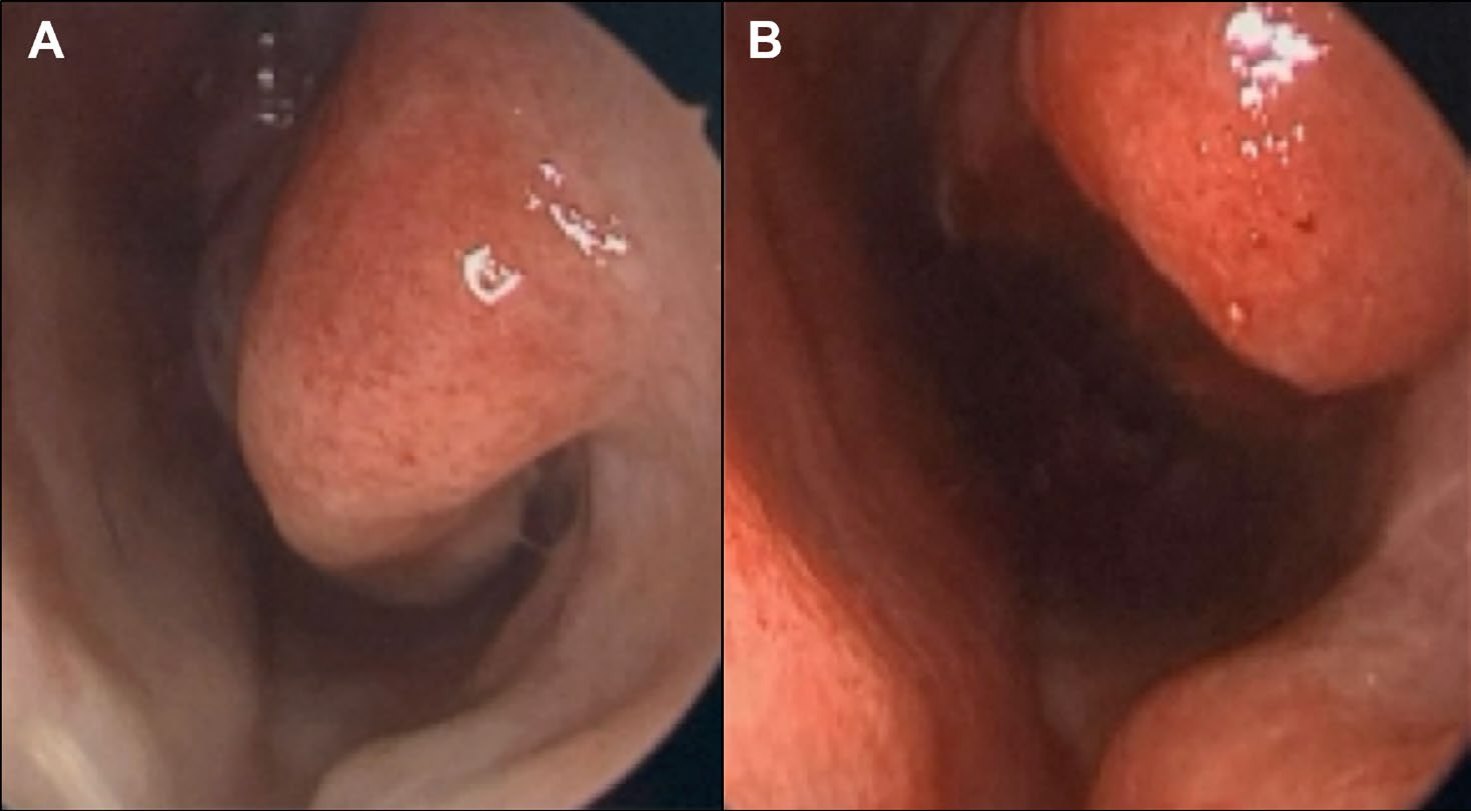
Endoscopic images of the nasal cavity before (A) and after (B) inferior turbinate reduction, demonstrating decreased turbinate size and improved airway visualization.

A 14‐year‐old female patient presented with chief complaints of reduced smell and nasal congestion. Although she had contracted COVID‐19 two years prior, her olfactory symptoms had only developed within the past year. Additionally, she experienced allergic symptoms such as frequent sneezing and rhinorrhea, and occasional headache. She was initially treated medically with cetirizine (Zyrtec) and fluticasone propionate (Flonase) daily. Persistent symptoms and physical exam findings of inferior turbinate hypertrophy led to consideration for surgical interventions (Figure 1). The patient elected to undergo bilateral inferior turbinate trim and adenoidectomy.

Two weeks postoperatively, the patient continued to experience anosmia, although she noted improvement in nasal congestion. At her two‐month postoperative follow‐up, her sense of smell was still subjectively absent despite adequate healing from surgery and daily saline rinses. Given her persistent olfactory dysfunction, her sense of smell was evaluated using the U‐Sniff variant of the Sniffin' Sticks odor identification test (SS‐OIT), a 12‐odor identification‐only subtest of the comprehensive Sniffin' Sticks battery [6], which revealed a diminished sense of smell (score 4 out of 12).

## 3 Differential Diagnosis, Investigations and Treatment

**FIGURE 2:**
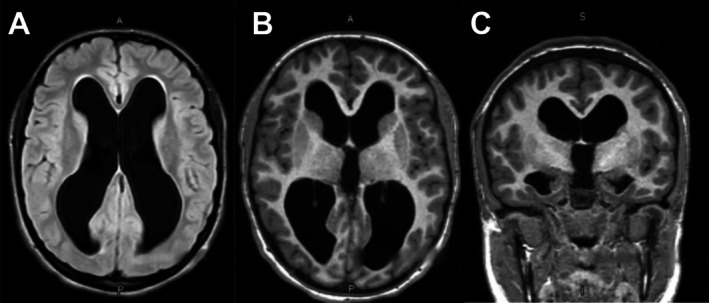
Preoperative axial (A and B) and coronal (C) MRI slices showing symmetrical dilation of the lateral and third ventricles with associated parenchymal thinning, consistent with noncommunicating hydrocephalus.

To further investigate the cause of her persistent hyposmia, magnetic resonance imaging (MRI) was obtained to evaluate for structural abnormalities of the olfactory bulbs or other components of the olfactory tract, as well as intracranial pathology (Figure 2).

A multiplanar multisequence MRI of the brain without contrast did not reveal any olfactory bulb abnormalities, but did demonstrate cerebral aqueduct obstruction with enlargement of the third and lateral ventricles. The imaging also showed low‐lying cerebellar tonsils with crowding at the foramen magnum. These findings indicated longstanding hydrocephalus secondary to stenosis of the cerebral aqueduct [7], along with thinning of the cerebral parenchyma. No evidence of syringomyelia was found. Soon after diagnosis, the patient underwent endoscopic third ventriculostomy.

## 4 Conclusion and Results (Outcome and Follow‐Up)

**FIGURE 3:**
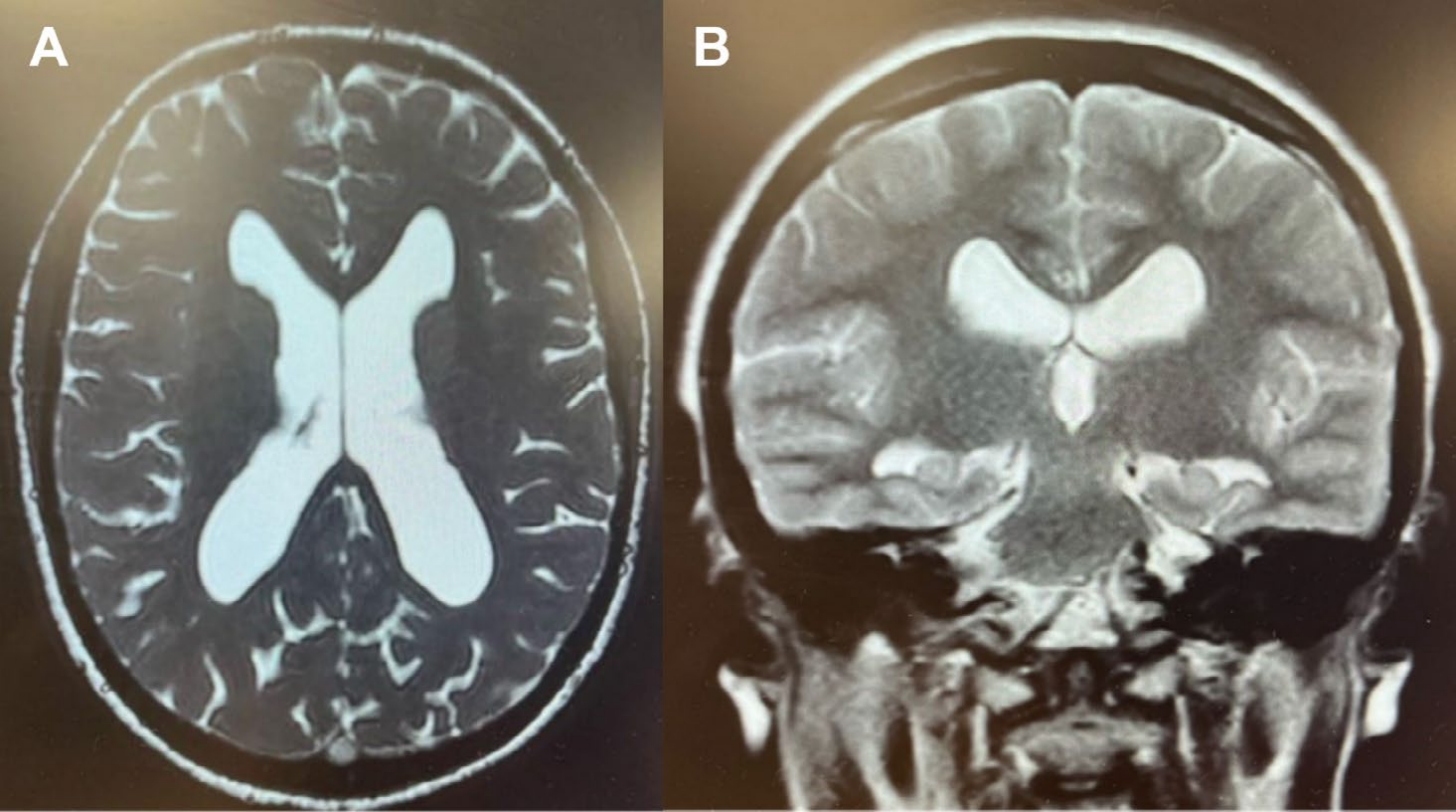
Postoperative axial (A) and coronal (B) MRI slices demonstrating reduced ventricular size following endoscopic third ventriculostomy.

The procedure resolved her reduced sense of smell and headaches and reversed her hydrocephalus (Figure 3). Her post‐operative Sniffin' Sticks score was 13/14 (normal). The constant nasal congestion has also improved significantly in the months following the nasal surgery.

## 5 Discussion

Olfactory dysfunction is an uncommon presenting symptom of noncommunicating hydrocephalus, but similar cases have been documented in the literature. In a report by Caminiti et al., olfactory dysfunction was evaluated with Sniffin' Sticks in a patient with gait disturbance, postural instability, and reported loss of smell. In that report, the Sniffin' Sticks score (2/12) and absent olfactory event‐related potentials (OERPs) were consistent with hydrocephalus induced anosmia [2]. Similarly, Yamazaki et al. [5] reported a case of noncommunicating hydrocephalus with associated anosmia, treated successfully with MRI‐guided diagnosis and endoscopic third ventriculostomy. The patient in their study also presented primarily with gait disturbance and anosmia. After surgical intervention, the patient experienced improvement in both motor and sensory symptoms, including the resolution of anosmia. Passler et al. [8] demonstrated reduced odor identification scores in patients with normal pressure hydrocephalus compared with healthy controls using the University of Pennsylvania Smell Identification Test (UPSIT). Podlesek et al. [9] reported reduced olfactory bulb volume on MRI in patients with idiopathic normal pressure hydrocephalus and demonstrated associated olfactory identification deficits using the Sniffin' Sticks test, supporting both structural and functional olfactory involvement in this hydrocephalus subtype.

Olfactory bulb morphology was normal on MRI in this patient. We hypothesize that chronic ventricular enlargement and elevated intracranial pressure in noncommunicating hydrocephalus may instead impair central olfactory processing through distortion of olfactory tracts or orbitofrontal pathways, resulting in a reduced sense of smell that can improve following cerebrospinal fluid diversion. Olfactory function includes multiple domains, such as odor detection threshold, discrimination, and identification. While olfactory identification deficits have been described in hydrocephalus, the extent to which hydrocephalus affects odor threshold and discrimination is unclear and has not been consistently characterized in the literature [2, 10].

Smell identification tests, such as the U‐Sniff 12‐odor variant of the Sniffin' Sticks Test used in this case, or the University of Pennsylvania Smell Identification Test, are reliable methods in identifying olfactory dysfunction [11]. However, imaging modalities are equally important in identifying anatomic etiologies of persistent olfactory dysfunction. Structural conditions such as noncommunicating hydrocephalus, ranging from classic cases of aqueduct stenosis to rarer entities such as cerebral arachnoid cysts, can be easily diagnosed with MRI. For post‐traumatic anosmia, functional MRI (fMRI) and SPECT/CT have been utilized and have been effective in identifying reduced activation of the orbitofrontal cortex [12]. In cases of post‐viral anosmia, Kim et al. [13] utilized positron emission tomography scans in identifying metabolic changes in the orbitofrontal cortex.

This case highlights anosmia as an atypical presenting symptom of noncommunicating hydrocephalus, emphasizing the importance of comprehensive diagnostic approaches. When hydrocephalus is identified in anosmic patients, surgical intervention such as endoscopic third ventriculostomy can effectively address olfactory dysfunction and other related symptoms. Appropriate imaging should be strongly considered in cases of unresolved olfactory dysfunction despite addressing common causes such as nasal obstruction. A multi‐modal approach, integrating clinical presentation, imaging, and sensory testing, is critical for diagnosing and managing persistent olfactory dysfunction.

## Author Contributions

**Bartels Walker:** data curation, investigation, writing – original draft. **Siddharth A. Patel:** data curation, methodology, writing – review and editing. **Caitlin Jackson:** data curation, methodology, project administration. **Graham M. Strub:** conceptualization, project administration, supervision, writing – review and editing.

## Funding

The authors have nothing to report.

## Ethics Statement

The authors have nothing to report.

## Consent

The patient included in this report provided written consent for the use of their intraoperative photographs.

## Conflicts of Interest

The authors declare no conflicts of interest.

## Data Availability

Data sharing not applicable to this article as no datasets were generated or analysed during the current study.
